# Association between handgrip strength and bone mineral density of Brazilian children and adolescents stratified by sex: a cross-sectional study

**DOI:** 10.1186/s12887-021-02669-1

**Published:** 2021-04-28

**Authors:** Bruna Thamyres Ciccotti Saraiva, Ricardo Ribeiro Agostinete, Ismael Forte Freitas Júnior, Daniel Eduardo Rodrigues de Sousa, Luis Alberto Gobbo, William Rodrigues Tebar, Diego Giulliano Destro Christofaro

**Affiliations:** Department of Physical Education, São Paulo State University (UNESP), School of Sciences and Technology, Presidente Prudente, SP CEP 19060-900 Brazil

**Keywords:** Adolescent, Bone density, Child, Health, Strength

## Abstract

**Background:**

To examine the association of handgrip strength (HGS) and bone mineral density (BMD) of Brazilian children and adolescents.

**Methods:**

The sample included 243 children and adolescents aged from 4 to 15 years (9.3 ± 2.2 years), 171 males and 72 females. The following measurements were performed: weight, height, trunk length, and years to the peak height velocity (PHV). The percentage lean soft tissue (PLST), percentage fat mass (PFM), and BMD were obtained using Dual Energy X-ray Absorptiometry (DXA) and HGS using a dynamometer.

**Results:**

In girls, HGS was positively associated with higher BMD, even after the adjustments, by arms [β = 0.006; *p* < 0.001], legs [β = 0.014; *p* < 0.001], pelvis [β = 0.019; p < 0.001], trunk [β = 0.013; p < 0.001], spine [β = 0.013; *p* = 0.008], and total body [β = 0.009; p < 0.001]. The same occurred in the boys, even after the adjustments a positive relationship was observed, whereas higher HGS was related to greater BMD in arms [β = 0.006; p < 0.001], legs [β = 0.017; p < 0.001], pelvis [β = 0.014; p < 0.001], trunk [β = 0.009; p < 0.001], spine [β = 0.008; p < 0.001], and total body [β = 0.007; p < 0.001].

**Conclusion:**

HGS was positively associated to BMD in boys and girls, regardless of age, PHV, PLST, and PFM.

## Introduction

Bone mineral density is defined as the bone mineral mass per unit area in grams by square centimeters, and the bone mineral content is the mineral mass component of bone in the form of hydroxyapatite, commonly measured in grams [1]. During childhood and, mainly, adolescence the greatest accumulation of bone mineral density occurs, with up to 35.8% of bone mineral content acquired 2 years before and 2 years after the peak height velocity [2, 3]. High accumulation of bone mass in this group is considered as a protective factor throughout life for development of diseases related to bone metabolism, such as osteopenia and osteoporosis [4]. Considering that early developmental life is an important period for laying down the foundation for life-long bone mineral density, studies focusing on extrinsic factors that contribute to bone mass (i.e., muscle mass) in this period are needed.

Regarding the effects on muscle tissue, several studies investigating sports practice or resistance training during growth showed a positive relationship between muscle mass and bone health [5]. More specifically, for muscle tissue variables, muscle strength has been considered an important predictor of muscular responses [6]. For this reason, it has received great attention in studies investigating the association with bone tissue variables, such as bone mineral density. In fact, because of its low cost, and easy and fast application, handgrip strength has been widely used as a method of muscle strength analysis. In adults, the usefulness of handgrip strength has been confirmed in studies that show lower handgrip strength associated with lower bone mineral density, mainly in women [7], and a high incidence of fractures in older adults [8].

In children and adolescents, muscle strength also seems to be associated with bone health [9]. In general, this relationship can be explained by the mechanostat that muscular activity generates tension in bones (i.e., exerting traction forces), resulting in adaptations in the bone tissue, recruitment of bone formation cells, and improvements in muscular strength [9, 10]. A recent systematic review of Torres-Costoso (2020) [9] showed that muscular strength of the upper limbs presented high association with bone variables than lower limb strength in children and young adults. Of note, this finding supports the measurement of handgrip strength as a method of muscle strength analysis. Lastly, the systematic review [9] did not identify any studies in pediatric populations from Brazil using methods to analyze muscle strength of upper limbs and bone variables.

Thus, the objective of this study was to analyze the association between handgrip strength and total body and segmental bone mineral density (upper limbs, lower limbs, spine, pelvis, and trunk) of Brazilian children and adolescents stratified by sex.

## Methods

This cross-sectional study was conducted in the city of Presidente Prudente, São Paulo, Brazil and was approved by the Ethics and Research Committee of the Institution responsible for this study (CAAE: 26702414.0.0000.5402, Opinion number: 549.549, approved on March 7, 2014). The study was carried out in partnership with a Philanthropic Institution, where the sample was recruited in 2015 and 2016.

The institution serves nearly 200 children and adolescents per year. The purpose of this  Institution are to ensure adequate care for children and adolescents of both sexes, especially basic protection, through services of coexistence, in the city of Presidente Prudente. We recruted all children and adolescents of this institution for evaluations. Those who refused to participate were not included in the study.

The minimum sample size was estimated by a minimum correlation of r = 0.44 for girls and r = 0.38 for boys between the HGS and total body BMD [11], a statistical power of 80%, and an alpha error of 5%. Thus, sample size was estimated to be 93 subjects, 53 boys and 40 girls. The initial convenience sample consisted of 309 children and adolescents. Of these, sixty-six were excluded from the analyses because they did not have a DEXA assessment (*n* = 24), did not have a handgrip strength assessment (*n* = 40), had articular problems (i.e., arthritis) (*n* = 1), and were pregnant (n = 1). Thus, the final sample consisted of 243 children and adolescents, aged 4 to 15 years (9.3 ± 2.2 years), 171 males and 72 females. The following inclusion criteria were adopted: 1) aged between 4 and 15 years; 2) enrolled in the Philanthropic Institution; 3) presenting an informed consent form signed by parents and/or guardians. The exclusion criteria of this study were: 1) pregnancy; 2) presenting any articular problems previously diagnosed by a physician.

### Anthropometric measurements

Body mass was measured using a digital scale (Filizzola PL 150, model Filizzola Ltda, Brazil), with a precision of 0.1 kg. Height was measured using a fixed stadiometer (Sanny, American Medical model of Brazil Ltda, Brazil) with accuracy of 1 mm according to standardized techniques described in the literature [12]. The sitting height was calculated utilizing a bench with a specific height of 50 cm and a fixed stadiometer (Sanny, American Medical model of Brazil Ltda, Brazil). After subtracting the length of the bench from the sitting height measurement (i.e., sitting height *minus* 50 cm), the trunk length was calculated considering the neck and head of the participant. Lastly, the leg length was calculated by subtracting the trunk length from total height (i.e., height *minus* trunk length). All anthropometric measurements were performed by two trained researchers with experience.

### Somatic maturation

Somatic maturation was estimated considering the years from the Peak Height Velocity, using the equation adopted by Mirward et al. (2002) [13]. The mathematical formula considers the anthropometric variables (i.e., body mass, height, and trunk and leg length) to obtain the remaining time for the adolescent to reach the Age of Peak Height Velocity (APHV). When considering the large variation in sample age, only the remaining time to reach PHV was considered in the analyses.

### Bone mineral density and body composition

Variables of bone mineral density (BMD-g/cm^2^), lean soft tissue, and fat mass were measured using Dual Energy X-Ray Absorptiometry (DEXA, Lunar DPX-NT; General Electric Healthcare, Little Chalfont, Buckinghamshire, UK). The exam performed refers to total body scans, which were stratified by body segment (upper limbs, lower limbs, trunk, spine, and pelvis) using the software of the equipment itself (GE Medical System Lunar, version 4.7). Scanner quality was tested by a trained researcher before the first exam of each day, following the manufacturer’s guidelines. All scans were performed in the physical assessment laboratory of the University in a controlled temperature room, by two trained researchers. The precision of the equipment related to the coefficient of variation was 0.66% considering the total body BMD of 30 subjects not involved in the study sample.

The values of lean soft tissue and fat mass were used in percentages in the analyses, with the aim of reducing the differences in body composition evident in samples with different ages and maturation stages [14], relativizing the values individually. Thus, the variables percentage lean soft tissue (PLST) and percentage fat mass (PFM) were created.

Lastly, each specific body segment (upper limbs, lower limbs, trunk, spine, and pelvis) was determined after the scan, by the same researcher. The region of interest (ROIs) was set in the enCORE software considering the anatomical points, as described below:

1. Arms were measured considering the position of the line passing through the upper edge of the acromial extremity of the clavicle and the separation of the trunk considering the midline of the glenohumeral joint.
2. Legs were measured considering the position of the line passing through the lower edge of the ischium.
3. The vertebral column was measured from the posterior-superior edge of the iliac crest (L4/L5 level) to the lower edge of the chin. The lateral cut was positioned as close as possible to the spine.
4. The pelvis was measured from the lower edge of the ischium to the posterior-superior border of the iliac crest (L4/L5 level).
5. The trunk was measured from the upper edge of the sternoclavicular joint to posterior-superior edge of the iliac crest (without head and neck). The lateral lines were performed considering the glenohumeral joint from a midline between trunk and arms.
6. Total BMD was obtained considering all body regions from the cranial vertex to the distal phalanx of the feet.

### Handgrip strength

Handgrip strength (HGS) was measured in kilograms (kg) using an electronic dynamometer (CAMRY Dynamometer, model EH101, Guangdong, China). The device was calibrated frequently. The following measurement procedures were adopted: 1) participants remained seated; 2) with the elbow flexed at 90 degrees; 3) participants held the dynamometer first with the right hand and then with the left hand; 4) they were required to hold and squeeze the dynamometer twice with each hand as hard as possible, with a 1-min interval between measures; 5) evaluators gave encouragement during the effort; 6) participants were required to squeeze the dynamometer for 3 to 5 s. The measurements were recorded by two experienced researchers. The mean handgrip strength was calculated from the values of the two grips.

### Statistical analysis

Descriptive data were calculated using mean and standard deviation stratified by sex. Kolmogorov-Smirnov normality test was performed to analyze data distribution. Comparisons between sexes were performed using the t-test for independent samples. Pearson’s correlations were performed to investigate the relationship between the dependent variable (i.e., BMD) and independent variable (i.e., HGS). Linear regression analysis was performed to verify the association between HGS and BMD stratified by sex. For this analysis, BMD was considered the dependent variable and HGS the independent variable, and the models were separately adjusted for age, years to PHV, PLST, and PFM. The magnitude of the correlations was interpreted as very strong from 0.9, strong from 0.7 to 0.9, moderate from 0.5 to 0.7, weak from 0.3 to 0.5, and negligible from 0 to 0.3 [15]. Statistical analyses were performed in Statistical Package for the Social Sciences (SPSS) software, version 15.0, and the statistical significance was set as a *p*-value lower than 0.05.

## Results

No differences in the anthropometric and maturation variables between the sexes. However, in the analysis of bone variables, girls presented higher values of BMD pelvis, BMD trunk, and BMD spine. The difference of PLST and PFM between boys and girls was observed but no differences in HGS between both sex (Table 1). When correlations between HGS and total and segmental BMD were evaluated, moderate to high (0.60 to 0.80) positive correlation were observed (Table 2).

**Table 1:** Characteristics of the sample and comparisons according to sex based on the independent t-test

Variable	Boys (***n*** = 171) Mean (SD)	Girls (***n*** = 72) Mean (SD)	***P***-value
Age (years)	9.36 (2.30)	9.39 (1.97)	0.923
Weight (kg)	38.97 (14.28)	40.90 (14.33)	0.338
Height (cm)	140.63 (13.92)	141.42 (13.32)	0.684
PHV (years)	−4.52 (1.35)	− 4.29 (1.18)	0.210
APHV (years)	13.18 (1.35)	13.65 (0.92)	0.313
HGS (kg)	16.04 (6.17)	16.16 (6.35)	0.894
PLST (%)	71.51 (13.93)	67.04 (11.39)	0.039*
PFM (%)	23.21 (11.92)	30.28 (13.50)	< 0.001*
BMD Arms (g/cm^2^)	0.63 (0.06)	0.63 (0.06)	0.961
BMD Legs (g/cm^2^)	0.93 (0.15)	0.95 (0.13)	0.354
BMD Pelvis (g/cm^2^)	0.87 (0.13)	0.91 (0.16)	0.023*
BMD Trunk (g/cm^2^)	0.74 (0.08)	0.77 (0.10)	0.039*
BMD Spine (g/cm^2^)	0.78 (0.10)	0.85 (0.18)	0.002*
BMD Total (g/cm^2^)	0.92 (0.08)	0.94 (0.08)	0.248

SD = standard deviation, PHV = years from peak height velocity, APHV = age of peak height velocity, HGS = handgrip strength, PLST = percentage lean soft tissue, PFM = percentage fat mass, BMD = bone mineral density, * = *p*-value < 0.05

**Table 2:** Pearson correlation between handgrip strength and BMD in children and adolescents (*n* = 243)

	Handgrip Strength	
	R	P-value
**Boys (n = 171)**		
BMD Arms (g/cm^2^)	**0.725**	≤0.001*
BMD Legs (g/cm^2^)	**0.771**	≤0.001*
BMD Pelvis (g/cm^2^)	**0.735**	≤0.001*
BMD Trunk (g/cm^2^)	**0.720**	≤0.001*
BMD Spine (g/cm^2^)	**0.604**	≤0.001*
BMD Total (g/cm^2^)	**0.685**	≤0.001*
**Girls (n = 72)**		
BMD Arms (g/cm^2^)	**0.764**	≤0.001*
BMD Legs (g/cm^2^)	**0.775**	≤0.001*
BMD Pelvis (g/cm^2^)	**0.795**	≤0.001*
BMD Trunk (g/cm^2^)	**0.801**	≤0.001*
BMD Spine (g/cm^2^)	**0.493**	≤0.001*
BMD Total (g/cm^2^)	**0.797**	≤0.001*

*BMD* bone mineral density, * = *p*-value< 0.05

The association between HGS and total and segmental BMD by sex was investigated using linear regression analyses. In females, higher HGS was related to higher BMD in all body regions, which remained significant after adjustments considering the effect of age and years to PHV, in the arms, legs, pelvis, trunk, and total body regions. However, after controlling for PLST and PFM, the associations returned to demonstrating significance in all body regions (Table 3). For boys, the relationships remained the same, higher HGS was related to higher BMD, even after adjusting for confounding factors, age, and years to PHV, as well as for PLST and PFM, in all body regions (Table 4).

**Table 3:** Relationship between handgrip strength and BMD in girls (*n* = 72)

Handgrip Strength									
	Unadjusted			Adjusted for age and PHV			Adjusted for PLST and PFM		
	β	CI (95%)	***P***-value	β	CI (95%)	***P***-value	β	CI (95%)	***P***-value
**Girls**									
BMD Arms (g/cm^2^)	**0.008**	**0.007; 0.010**	≤0.001*	**0.005**	**0.003; 0.008**	≤0.001*	**0.006**	**0.004; 0.009**	≤0.001*
BMD Legs (g/cm^2^)	**0.017**	**0.014; 0.020**	≤0.001*	**0.009**	**0.004; 0.014**	≤0.001*	**0.014**	**0.010; 0.019**	≤0.001*
BMD Pelvis (g/cm^2^)	**0.020**	**0.017; 0.024**	≤0.001*	**0.015**	**0.010; 0.021**	≤0.001*	**0.019**	**0.014; 0.025**	≤0.001*
BMD Trunk (g/cm^2^)	**0.013**	**0.011; 0.016**	≤0.001*	**0.010**	**0.006; 0.014**	≤0.001*	**0.013**	**0.010; 0.016**	≤0.001*
BMD Spine (g/cm^2^)	**0.014**	**0.008; 0.020**	≤0.001*	0.006	−0.003; 0.016	0.165	**0.013**	**0.004; 0.022**	0.008*
BMD Total (g/cm^2^)	**0.010**	**0.008; 0.013**	≤0.001*	**0.006**	**0.003; 0.009**	≤0.001*	**0.009**	**0.006; 0.012**	≤0.001*

*PLST *percentage lean soft tissue, *PFM* percentage fat mass, *PHV* years from peak height velocity, *CI* confidence interval, *BMD* bone mineral density, * = *p*-value< 0.05. Variance Inflation Factor values for collinearity statistics: Handgrip Strength = 2.428; Age = 7.517; PHV = 6.804; PLST = 5.982; PFM = 4.771. *p < 0.05

**Table 4:** Relationship between handgrip strength and BMD in boys (*n* = 171)

Handgrip Strength									
	Unadjusted			Adjusted for age and PHV			Adjusted for PLM and PFM		
	β	CI (95%)	***P***-value	β	CI (95%)	***P***-value	β	CI (95%)	***P***-value
**Boys**									
BMD Arms (g/cm^2^)	**0.007**	**0.006; 0.008**	≤0.001*	**0.005**	**0.003; 0.006**	≤0.001*	**0.006**	**0.004; 0.007**	≤0.001*
BMD Legs (g/cm^2^)	**0.019**	**0.017; 0.021**	≤0.001*	**0.009**	**0.006; 0.013**	≤0.001*	**0.017**	**0.014; 0.020**	≤0.001*
BMD Pelvis (g/cm^2^)	**0.016**	**0.014; 0.019**	≤0.001*	**0.010**	**0.006; 0.013**	≤0.001*	**0.014**	**0.011; 0.017**	≤0.001*
BMD Trunk (g/cm^2^)	**0.010**	**0.009; 0.012**	≤0.001*	**0.007**	**0.005; 0.010**	≤0.001*	**0.009**	**0.007; 0.010**	≤0.001*
BMD Spine (g/cm^2^)	**0.010**	**0.008; 0.012**	≤0.001*	**0.009**	**0.006; 0.013**	≤0.001*	**0.008**	**0.006; 0.010**	≤0.001*
BMD Total (g/cm^2^)	**0.010**	**0.008; 0.011**	≤0.001*	**0.006**	**0.003; 0.008**	≤0.001*	**0.007**	**0.005; 0.010**	≤0.001*

*PLST *percentage lean soft tissue, *PFM* percentage fat mass, *PHV* years from peak height velocity, *CI* confidence interval, *BMD* bone mineral density. Variance Inflation Factor values for collinearity statistics: Handgrip Strength = 2.577; Age = 6.290; PHV = 3.882; PLST = 2.505; PFM = 2.697. * *p* < 0.05

## Discussion

This study aimed to analyze the relationship between HGS and total and segmental BMD. The main finding was the positive relationship between these variables regardless of sex and after adjusting for confounding factors such as age and somatic maturation, as well as for PLST and PFM.

In the current study, the significant relationship between HGS and arms, legs, pelvis, trunk, spine, and total BMD may be explained by the high composition of trabecular bone in these anatomical regions [16]. In addition, handgrip strength has been related to total muscle strength [17], and muscle strength has been related to higher moderate-to-vigorous intensity physical activities in youth [18, 19]. This may provide higher impact and remodeling stimulation in these sites due to mechanical load, which may be even higher in boys as they are traditionally more physically active than girls [20]. However, the current study did not assess the physical activity level to confirm this hypothesis. In girls, the HGS showed a significant relationship with most bone variables, including arms, legs, pelvis, trunk, and total body BMD. This relationship in girls can be explained by the peak of bone accumulation, which occurs from 11 to 14 years in girls and slightly later in boys (i.e., from 13 to 16 years) [21]. These observations corroborate the findings that the relationship between HGS and BMD in girls, even after adjustments, was greater considering the leg, pelvis, trunk, and total body, whereas in boys the relationship remained the same.

Our findings corroborate the recent systematic review by Torres Costoso (2020) [9] which found that muscular strength of upper limbs is associated with bone mineral density and can also be considered as a marker of skeletal health during growth in different populations including Brazilian children and adolescents. Similar to previous studies [11, 22], the current study also observed a significant relationship between HGS and hip and trunk BMD in boys and girls. Nasri et al. (2013) [23] found positive correlations between the practice of combat sports and variables of BMD in 50 adolescents when compared to a group of 30 sedentary adolescents. The study indicated that the HGS was higher in the athlete group and that the HGS of the dominant arm was associated with the total body and leg bone density. The HGS of the non-dominant arm was strongly related to the density of the spine, as well as being related to the density of the arms and legs, and it was shown to be a better predictor of bone density in the analyzed group. Although Nasri et al. (2013) [23] did not stratify and adjust by sex, the findings considering HGS as a predictor of bone density were similar to our study.

The present study advances the literature through adjustments in the models using somatic maturation to give more precise results because of the different biological maturation stage of individuals. The maturational stage may present differences in performance [24] and affect the body composition and bone tissue [14, 25], especially in circumpubertal years (i.e., between − 2 and + 2 years from the PHV) [21], a period when 39% of young-adult total body bone mass is accrued [25]. In fact, this period is composed of great hormonal releases such as testosterone, estradiol, and insulin-like growth factor-1 (IGF-1), anabolic hormones active in the release of bone formation cells. In typical growth, more advanced maturational stages are related to greater bone density [26]. This fact is supported by findings of a previous study analyzing 199 individuals from 6 to 19 years, separated into subgroups, whereas a sudden increase in HGS was observed in male participants from 14 years because of the puberty period [27].

Regarding the adjustments of the analysis using PLST and PFM, the relationships between HGS and BMD observed in the unadjusted model, remained across all anatomical regions in boys and girls. These results demonstrate that although muscle strength is a measure of muscle performance which directly depends on the cross-sectional muscle area [28], and that some studies evidenced the effect of fat mass on BMD [29], the control of these relativized variables (percentage) does not seem to affect this association.

Bone density is affected by several factors, such as genes, nutrition, hormones, muscle tension, body composition, and impact stimuli from physical activity and exercise. A load applied to the bones through continuous muscle contractions affects the strength and geometry of bones. This tension applied in a chronic way together with activities that provide high impact, stimulates a decrease in bone resorption, and an increase in bone formation in the specific area exposed to the load, which results in gains in bone density [30–32]. Therefore, the level of muscle tension which an individual is able to exert may be indicative of their state of bone density.

In the current study, we must also consider that the period of menarche in girls may justify the stronger relationship between HGS and BMD as it represents the beginning of significant bone mass gain and occurs earlier in girls than boys. However, a study to analyze the relationship between HGS and BMD at the beginning of the period of menarche is needed to clarify this possibility [26, 33]. The current study included analyses of BMD correlated with HGS in children and adolescents, which most studies only address in adult and older adult populations, motivating the debate on the best strategies for gains in BMD, especially in children and adolescents. Therefore, considering the ease of measuring HGS in a pediatric population and its significant association with BMD, this technique should be more commonly incorporated into studies investigating bone density in young adults.

Despite the relevance, the present study presents some limitations, such as the study design, which is cross-sectional, so no cause and effect can be indicated, and the dynamometer which was not adjusted to accommodate smaller hand sizes. Another limitation is related to the handgrip strength test which could not estimate muscle strength of different body sites in the same form as in the arms (specifically hands) to infer the direct relationship of mechanical load for bone remodeling in the different body regions. In addition, there was a lack of food control, since foods rich in vitamin D, calcium, and proteins could also have influenced our results. The lack of analysis of genetic profiles limited the adjustment for potential confounding factors, such as ACE I-II polymorphisms and other gene variations, which could moderate the relationship between muscle strength and BMD. In addition, the lack of control of habitual physical activity and biochemical analyses (bone markers) are also considered limitations of the study. Meanwhile, the use of a gold standard assessment for BMD, the sample size, and the sex-stratified analysis were strengths of the study, as well as the adjustments for age, percentage lean soft tissue, percentage fat mass, and somatic maturation, due to the wide age range (i.e., between 4 to 15 years) with different maturational stages and the different proportions between sexes (171 boys versus 72 girls).

As practical applications of the study, the low cost of the HGS, an indicator of physical fitness, can be highlighted, which is associated with higher BMD values that requires equipment considered expensive. Considering the high incidence and prevalence of injuries due to bone fragility and the reduction in physical activity, care and monitoring of these variables is necessary during childhood and adolescence. In addition, the results of this study may help health care professionals by providing support on how to monitor these variables using a low-cost method.

## Conclusions

Considering the results of this study, greater handgrip strength is related to higher bone density in boys and girls, even after adjusting for age, somatic maturation,  percentage lean soft tissue, and percentage fat mass.

## Data Availability

The dataset of the study can be made available considering a justifiable request by contacting the first author.

## Abbreviations

BMD: Bone mineral density
HGS: Handgrip strength
PLST: Percentage lean soft tissue
PFM: Percentage fat mass
PHV: Peak height velocity
